# A novel triphenylacrylonitrile based AIEgen for high contrast mechanchromism and bicolor electroluminescence†

**DOI:** 10.1039/c7ra10174k

**Published:** 2018-01-02

**Authors:** Jiayou Hu, Bingli Jiang, Yongyang Gong, Yuanli Liu, Gang He, Wang Zhang Yuan, Chun Wei

**Affiliations:** Key Laboratory of New Processing Technology for Non-ferrous Metals and Materials, Ministry of Education, Guangxi Key Laboratory of New Energy and Building Energy Saving, College of Materials Science and Engineering, Guilin University of Technology Guilin 541004 China yygong@glut.edu.cn 1986024@glut.edu.cn; College of Pharmacy, Guilin Medical University Guilin 541004 China; School of Chemistry and Chemical Engineering, Shanghai Jiao Tong University Shanghai 200240 China wzhyuan@sjtu.edu.cn

## Abstract

A novel thermally stable and aggregation-induced emission (AIE) active compound, 2,2'-(([1,1′-biphenyl]-4,4′-diylbis(phenylazanediyl))bis(4,1-phenylene))bis(3,3-diphenylacrylonitrile) (BP2TPAN) was synthesized through a C–N coupling reaction between 2-(4-bromophenyl)-3,3-diphenylacrylonitrile (Br-TPAN) and *N*,*N*′-diphenyl-1,4-phenylenediamine, under mild conditions using Pd(OAc)_2_ and P(*t*-Bu)_3_ as a catalyst. The BP2TPAN was characterized by nuclear magnetic resonance spectroscopy, high resolution mass spectrometry and elemental analysis. The thermal analysis showed that the glass transition and decomposition temperatures (5% weight loss) are 96 and 414 °C, respectively. The fluorescent emission peaks changes at 540 and 580 nm upon grinding were attributed to a transformation from crystal to amorphous occurring by altering the condensed state. The photoluminescence quantum yield and fluorescence lifetime of the as prepared and ground samples were 74.3 and 8.4%, 3.4 and 5.1 ns, respectively. The difference of the luminous efficiency of before and after grinding samples indicates BP2TPAN has a high contrast more importantly, both doped and nondoped OLED devices emit different color, the doped one is highly efficient and its *L*_max_, CE_max_, PE_max_ and EQE are up to 15 070 cd m^−2^, 11.0 cd A^−1^, 7.5 lm W^−1^, and 3.1%, respectively.

## Abstract 2

An AIE luminogen with high contrast mechanochromic and bicolor electroluminescence.

## Graphical abstract

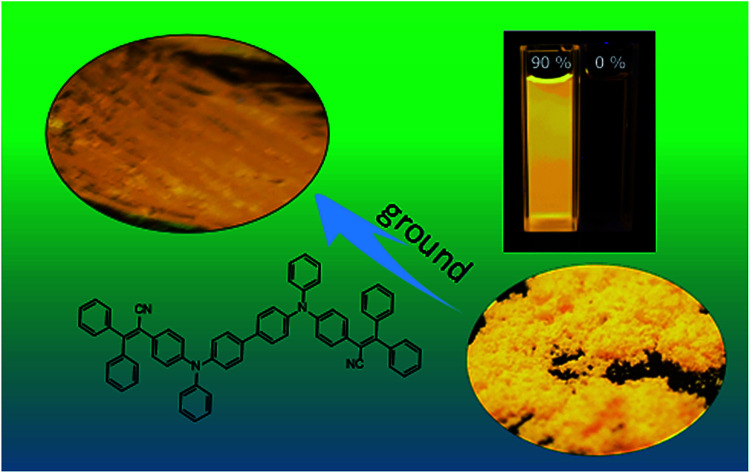

## 1 Introduction

Organic luminescent materials which show dynamically switchable solid-state emission in respect to mechanical stimuli such as shearing and grinding, have attracted increasing interest owing to their academic importance and promising applications in optical storage, mechanical sensors, displacement or deformation detectors, security paper, optical memory and optoelectronic devices.^1–9^ Efficient solid state emission is a key requirement for mechanochromic luminescent materials. However, conventional organic fluorescent molecules suffer from aggregation caused quenching (ACQ),^10^ which leads to poor solid state emission, and greatly restricts practical applications because luminescent materials are normally used in aggregated states, such as in OLEDs. Although intensive efforts have been made to reduce or completely eliminate the ACQ effect through chemical or physical methods, such as introduction of bulky alicyclics, encapsulation by surfactants, or blending with transparent polymers,^11,12^ these approaches would inevitably damage their optoelectronic properties. Fortunately, in 2001, Tang and co-workers discovered a novel phenomenon from twisted siloles, an aggregation-induced emission (AIE), which is opposite to the ACQ effect.^13^ Namely, these compounds are practically nonluminescent in solutions, but become highly emissive in aggregated states. The AIE active molecules are highly fluorescent in the solid state, which is an essential requirement for mechanochromic materials.

The mechanochromic phenomenon have been explained as an alteration of molecular packing modes, such as crystal to amorphous form transformation, formation of excimers/exciplex, or planarization under the action of forces.^14^ Over the past few years, many novel twisted conformations AIE active mechanochromic materials have been developed by Chi,^15^ Park,^16^ Tian,^17^ Tang,^18^*et al.* group, and the relationship between the molecular structure and the mehcanochromic luminescence has been evaluated.^14^ However, a few studies on AIE mechanochromic materials with its high thermal stability and high contrast have been reported.^19^ On the other hand, AIE compounds are ideal alternatives used for OLED applications.^20^ So far, however, many AIE compounds are mainly fabricated OLED devices with only one electroluminescent color.^21–23^ However, a few studies reported OLED devices with two or more electroluminescence emitting color based AIE compounds.^24,25^

**Scheme 1:**
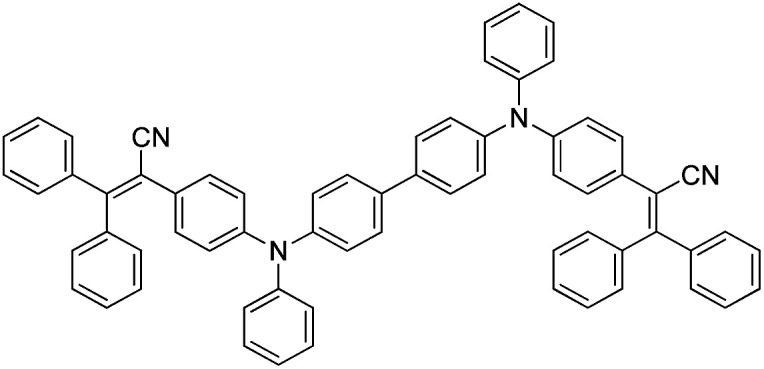
Chemical structure of BP2TPAN.

Herein, a novel highly twisted conformation compound BP2TPAN with good thermal stability, containing a triphenylacrylonitrile (TPAN), as depicted in Scheme 1, was successfully synthesized by C–N coupling between 2-(4-bromophenyl)-3,3-diphenylacrylonitrile (Br-TPAN) and *N*,*N*′-diphenyl-1,4-phenylenediamine (Fig. S1†).

BP2TPAN is AIE-active, and its photoluminescence quantum yield (PLQY) of as prepared samples (*Φ*_asp_) up to 74.3%. BP2TPAN solids also exhibit obvious mechanochromism behavior; upon grinding, the emission maxima of the as prepared powders were changed from 540 to 580 nm. It's also showed good thermal stabilities, with degradation temperature (*T*_d_, at which a sample loses 5% weight) and glass transition temperature (*T*_g_) being are 414 and 96 °C, respectively. More importantly, the doped and nondoped OLED devices show different emission color, which is rarely reported.

## 2 Experimental

### 2.1 Materials

Sodium *tert*-butoxide, sodium hydride (60 wt% in mineral oil), palladium diacetate, *N*,*N*′-diphenylbenzidine, were obtained from TCI (Shanghai) Development Co., Ltd. Benzophenone and 4-bromophenylacetonitrile were obtained from J&K chemical Scientific Ltd. Tri-*tert*-butylphosphine (0.49 M in toluene) was purchased from Puyang Huicheng Electronic Material Co., Ltd. Toluene was re-distilled under normal pressure from CaH_2_ under nitrogen immediately prior to used. THF was distilled from sodium benzophenone under nitrogen before used. The commercially available reagents were used without further purification.

### 2.2 Characterization

NMR spectrum was recorded on Bruker AMX-400 NMR spectrometer or Bruker AVANCE III HD600 600 MHz NMR instrument in deuterated solvent at room temperature. High-resolution mass spectrometry (HRMS) was performed on Bruker Daltonics ULTRAFLEXTREME MALDI-TOF/TOF mass spectrometer. UV-vis absorption and emission measurements were performed on a TU-1901 UV-vis spectrophotometer and a Perkin-Elmer LS 55 luminescence spectrometer, respectively. X-ray diffraction (XRD) measurements were carried out using Bruker D8 Advance TXS XRD with Cu Kα radiation at room temperature. Thermogravimetric analysis (TGA) and differential scanning calorimetric (DSC) measurements were conducted on TGA Q5000 V3.13 Build 261 instrument and Netzsch DSC 200 F3 under nitrogen at a heating and cooling rate of 5 °C min^−1^, respectively. Emission quantum yields (*Φ*_F's_) of BP2TPANs in solvents were estimated by using quinine sulfate (*Φ*_F_ = 54% in 0.1 N H_2_SO_4_) as standard, while solid-state efficiencies were determined using an integrating sphere. The ground-state geometries were optimized using the density functional with B3LYP hybrid functional at the basis set level of 6-31G(d). All calculation were performed using the Gaussian 09 package.

### 2.3 OLED fabrication

The device was fabricated by the following processes. First, ITO-coated glass substrates were cleaned successively using deionized water, acetone, and isopropanol in an ultrasonic bath and then dried in a drying cabinet followed by pretreatment with oxygen plasma. Then, the organic films of *N*,*N*,*N*′,*N*'-tetrakis(4-methoxy-phenyl)benzidine (MeO–TPD), 4,4′,4′′-tri(9-carbazoyl)triphenylamine (TCTA), AN2TPAN, 4,7-diphenyl-1,10-phenanthroline (Bphen), and 8-hydroxyquinolinatolithium (Liq) were deposited by thermal evaporation under a base vacuum of about 10^−6^ Torr. Finally, aluminum (Al) metal was evaporated in another vacuum chamber without breaking the vacuum. The thicknesses of the films were determined by quartz thickness monitors. The active area of the EL device, defined by the overlap of the ITO and the cathode electrode, was 3 mm × 3 mm. Current density–voltage and current efficiency–current density characteristics was measured with a computer controlled Keithley 2400 Source Meter and BM-7A Luminance Colorimeter. The electroluminescence spectrum was measured by a Labsphere CDS-610. All measurements were carried out under air at room temperature without device encapsulation.

### 2.4 Synthesis

#### Synthesis of 2-(4-bromophenyl)-3,3-diphenylacrylonitrile (Br-TPAN)

Into a 100 mL, two-necked, round bottom flask equipped with a condenser were placed 10 mmol (1.83 g) of benzophenone, 15 mmol (603 mg) of NaH (60 wt% in mineral oil), and 50 mL of toluene under nitrogen. The mixture was stirred at 80 °C for 10 min, then 11 mmol (2.17 g) of 4-bromobenzylnitrile in 10 mL of toluene was added drop-wise over 20 min while maintaining at 80 °C. The reaction was lasted for another 10 h, cooling to room temperature, after solvent evaporation, 100 mL water and 50 ml chloroform respectively, were added. The organic layer was collected and washed with brine three times. The organic phase was then dried over anhydrous MgSO_4_ and concentrated under vacuum. The crude product was purified by silica-gel column chromatography using petroleum ether/dichloromethane (DCM) as eluent (8/1–4/1). 3.05 g white solid was obtained in 84% yield. ^1^H NMR (400 MHz, CDCl_3_), *δ* (TMS, ppm): 7.43 (m, 5H), 7.34 (d, 2H), 7.29 (m, 1H), 7.22 (t, 2H), 7.14 (d, 2H), 7.01 (d, 2H). ^13^C NMR (100 MHz, CDCl_3_), *δ* (TMS, ppm): 158.41, 140.12, 138.72, 133.80, 131.70, 131.25, 130.67, 130.09, 129.87, 129.26, 128.49, 128.44, 122.55, 119.71, 110.43.

#### Synthesis of BP2TPAN

Into a 50 mL two-necked round bottom flask were placed 1 mmol (336 mg) *N*,*N*′-diphenyl-1,4-phenylenediamine, 2.5 mmol (900 mg) Br-TPAN, 3 mmol (288 mg) of *t*-BuONa, 10% mmol (22 mg) Pd(OAc)_2_, 0.5 ml of P(*t*-Bu)_3_ (0.45 M in toluene solution). The flask was evacuated under vacuum and flushed with dry nitrogen for three times. Then 30 mL fresh toluene was injected. Then the mixtures were heated to reflux under stirring for 36 h. Upon cooling to room temperature, the mixture was added 5 g silica (200–300 mesh), then, mixing uniform. Soxhlet extractor (250 mL) was placed 30 g silica (200–300 mesh), and the mixing mixture was added, the target compound BP2TPAN was extraction by 500 mL chloroform. Solvent was reduce evaporation to 100 mL, filtrate and filters wash with 20 mL chloroform three times, 621 mg yellow solid obtained in 69%. ^1^H NMR (600 MHz, THF-d_8_, *δ*) 7.58–7.39 (m, 14H, Ar-H), 7.32–7.02 (m, 26H, Ar-H), 6.98–6.88 (m, 4H, Ar-H), 6.68–6.58 (m, 2H, Ar-H); HRMS (MALDI, *m*/*z*): [M + H] calcd for C_66_H_46_N_4_, 894.37225; found 894.28727. Elemental analysis calcd (%): C, 88.65%; H, 5.18%; N, 6.26%; found: C 88.08%, H 5.33%, N 6.31%.

## 3 Results and discussion

### 3.1 Synthesis

BP2TPAN was synthesized according to the route shown in Fig. S1 (ESI†). First, Br-TPAN was synthesized according to the literature procedure through a Knoevenagel condensation reaction between 4-bromophenylacetonitrile and benzophenone.^26^ Then, the target molecule, desirable compounds of BP2TPAN, was obtained in good yields by Buchwald–Hartwig C–N cross-coupling reaction between Br-TPAN and *N*,*N*′-diphenyl-1,4-phenylenediamine, using Pd(OAc)_2_ and (*t*-Bu)_3_P as catalysts under mild conditions.^27^

BP2TPAN was characterized by standard spectroscopic methods, from which satisfactory analysis data corresponding to its molecular structures was obtained (see Fig. S2–4 ESI† for detail). For example, thin layer chromatography (TLC) plates indicated high purity of the isolated compounds in 1 : 1 petroleum ether–chloroform solvent mixture, as only a single spot was obtained for each compound (Fig. S2, ESI†). HRMS (Fig. S4 ESI†) peak [M]^+^ at *m*/*z* 894.28727 (calcd 894.37225) confirms the formation of expected adducts. Moreover, elemental analysis results of C 88.08%, H 5.33%, and N 6.31% are highly consistent with their theoretical values. Results altogether indicates that BP2TPAN was sufficiently pure.

### 3.2 AIE

**Fig. 1:**
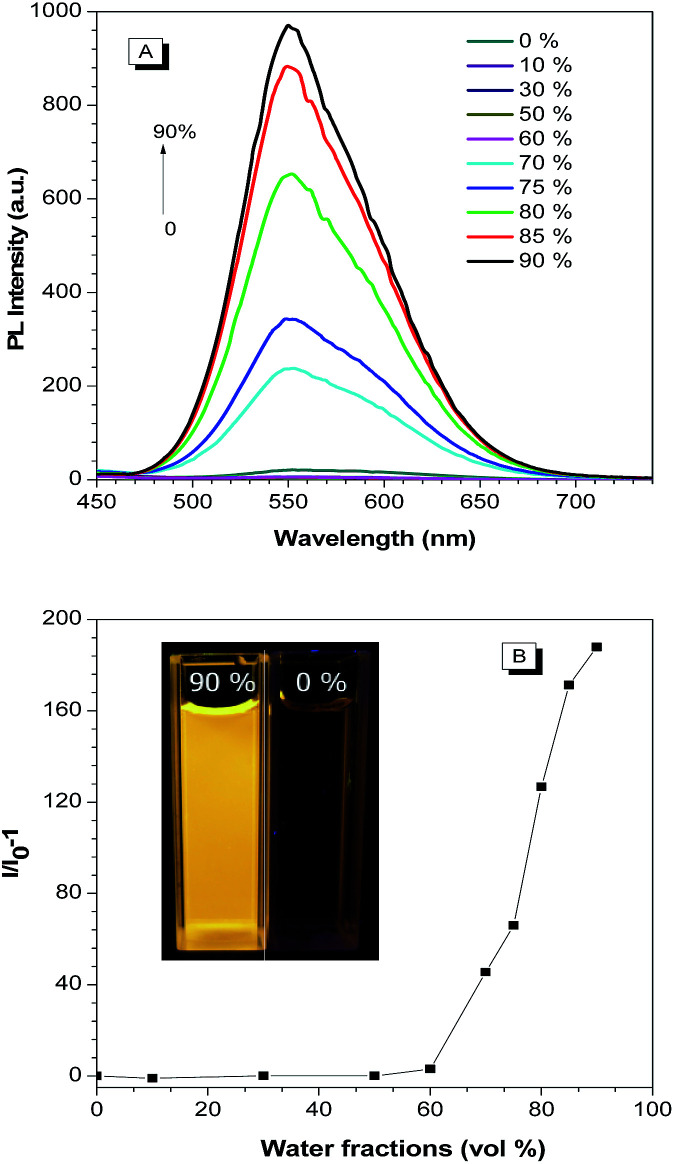
(A) PL spectra of BP2TPAN in THF and THF/water mixtures. (B) Plots of PL peak intensity *vs.* water fraction (*f*_w_). Concentration = 5 μM; excitation wavelength = 400 nm. The inset graphs are the solution of BP2TPAN in THF (*f*_w_ = 0%) and its suspensions in THF/water mixture with *f*_w_ = 90% under 365 nm UV light illumination.

Our previous work have confirmed that TPAN is a typical crystallization-induced emission luminogens,^28^ which could yielded new AIE activated compounds when combined with aniline, diphenylamine, triphenylamine and carbazole *et al.*^2,8,24,27,28^ It is therefore envisioned that BP2TPAN is also an AIEgen. BP2TPAN shows two absorption peaks at 340 and 394 nm in THF (Fig. S5, ESI†). The former is attributable to the π–π* transition, the latter corresponds to the intramolecular charge transfer (ICT) transition. To verify AIE activity of BP2TPAN, it was slightly solubilized in organic solvents (THF), no visible emission was observed. However, as prepared (Asp) solid powder, it emits strong yellow light at 540 nm with quantum efficiency of 74.3%, indicating that it is AIE-active. Emission spectra of BP2TPAN in THF and THF/water mixtures were also measured. Water was chosen because it is a typical nonsolvent for BP2TPAN, in which BP2TPAN molecules will be aggregated. When the water fraction (*f*_w_) is ≤60%, only weak signals were recorded, since the molecules were dissolved in the mixture. However, when *f*_w_ is increased to 70%, the emission intensity was swiftly boosted because of the molecular aggregation, giving a strong yellow emission at 560 nm (Fig. 1). Further addition of water continuously strengthened the emission intensity. In the 10 : 90 THF–water mixture, the emission was remarkably enhanced compared to that in THF (Fig. 1). The AIE nature of BP2TAPN also visible from the emission contrast of BP2TAPN in THF and 10 : 90 THF–water mixture (Fig. 1B). To quantitatively evaluate the AIE effect, PLQY of the luminogens in both solution (*Φ*_soult_) and as-prepared solid states were determined. The *Φ*_soult_ values of BP2TPAN in THF are as low as 0.1%, thus confirming that it is practically non-luminescent nature in solvents. However, the values for as prepared solids increased to 74.3%, indicating AIE characteristic.

**Fig. 2:**
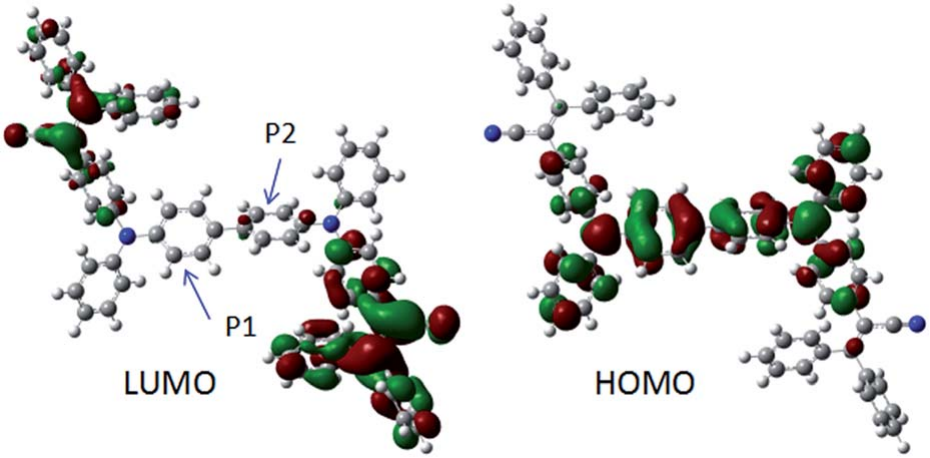
Optimized geometries and molecular orbital amplitude plots of HOMO and LUMO energy levels of BP2TPAN.

In order to better understand the nature of AIE and the geometry of BP2TPAN, quantum chemical optimization of their energy levels based on DFT/B3LYP/6-31G(d) was conducted. The optimized geometries and HOMO/LUMO plots of BP2TPAN are illustrated in Fig. 2. The molecule adopt highly twisted propeller-like non-planar conformations, which are favorable for active intramolecular rotations of multiple phenyls in solutions, thereby powerfully dissipating the excitons energy and making them non-emissive in solvents. When aggregated as suspensions, powders, thin solid films, on one hand, the intramolecular rotations are impeded; on the other hand, the propeller-like configurations prevent the formation of detrimental excimers or exciplexes, thereby generating boosted emissions. Notably, the dihedral angles of the adjacent phenyl rings (*P*1 and *P*2) is ≈20°, the non-planar conformations of biphenyl (*P*1–*P*2) decreased conjugated degree. Moreover, the electron clouds of the LUMO levels for BP2TPAN are mainly located on the electron-accepting TPAN peripheries; however, those of HOMO levels are dominated by orbitals from the electron-donating triphenylamine units. Generally, such electron distribution imparts an intrinsic intramolecular charge transfer property to dye molecules. This intramolecular charge transfer might balances hole and electron mobility in OLED devices and improve device performance.

### 3.3 Mechanochromic behavior

**Fig. 3:**
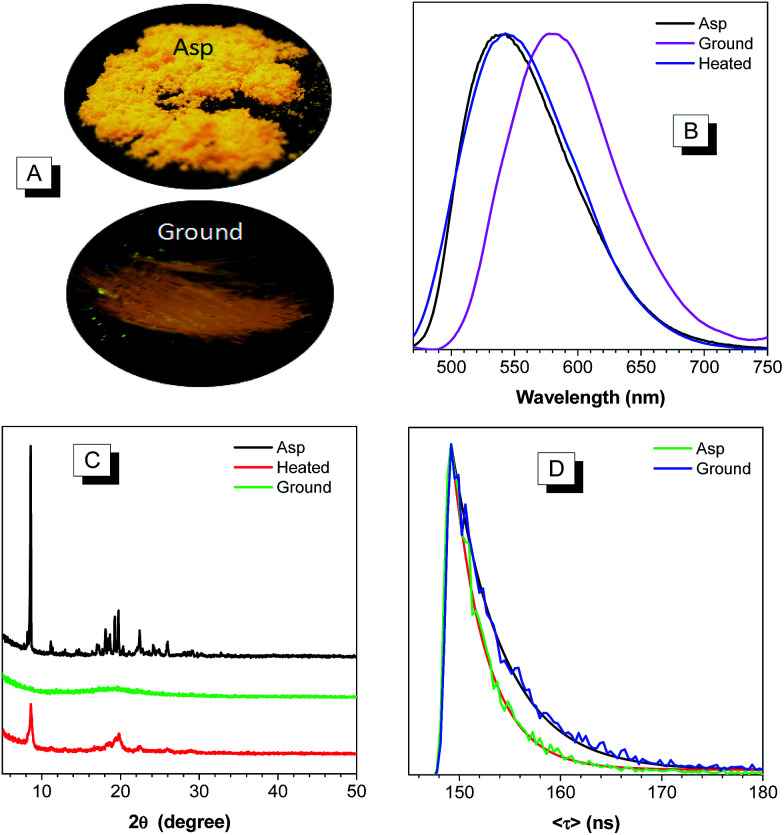
(A) The photographs of as prepared BP2TPAN and its ground samples under 365 nm UV irradiation. (B and C) Are emission spectra and XRD patterns of BP2TPAN under different condensed states. (D) Fluorescence lifetime of BP2TPAN and its ground sample.

The remarkably twisted conformations and high PLQY of BP2TPAN make it potentially promising as mechanochromic luminogen with a high contrast. Upon grinding in an agate mortar with a pestle, the emission color of the as prepared solid changes from yellow to orange (Fig. 3A), with its emission maximum red shifted from 540 to 580 nm and the (Fig. 3B). When the ground powder annealed at 120 °C under nitrogen for 10 min or fumed with chloroform vapor, the emission peaks restored to 542 nm (Fig. 3B).

To get more insights into the mechanism, XRD measurement of the as prepared, ground, and annealed samples were performed. As shown in Fig. 3C, the as prepared BP2TPAN display sharp and intense reflections at 2*θ* ≈ 8.6° and 19.5°, which indicate their well-defined crystalline order. However, the diffraction curves of the ground sample do not show any noticeable reflections in low intensity, indicating an amorphous structure. The amorphous sample, when annealed at 120 °C in N_2_ atmosphere, the diffraction peaks which coincided with those in the as prepared solids emerge again, indicating recovery of some crystalline order. These results also indicate that the interchange between crystalline–amorphous packing modes causes the mechanochromism effect in BP2TPAN.

Although mechanochromic materials with switchable emission colors are reported in the literature, use external force to manipulate the fluorescent quantum yield is seldom reported.^29^ For BP2TPAN, PLQY of its the ground sample is lower than those as prepared samples, decrease of PLQY from 74.3% to 8.4% was observed. This result is consistent with the results from the naked eye observation, and indicating BP2TPAN has high contrast. This phenomenon may be attributed to crystals that readily break, trigger planarization of molecular conformation under external pressure, as well as the formation of excimers. Theoretically, excimer formation would promote fluorescence lifetime and decrease photoluminescence quantum yield.^18^ Herein the fluorescence lifetime values of the as prepared and ground amorphous solids of BP2TPAN are determined as 3.4 and 5.1 ns (Fig. 3D), respectively. The increased lifetime and decrease photoluminescence quantum yield of the ground sample suggested ground samples forms of excimers upon mechanical stimuli.

### 3.4 Thermal stability

**Fig. 4:**
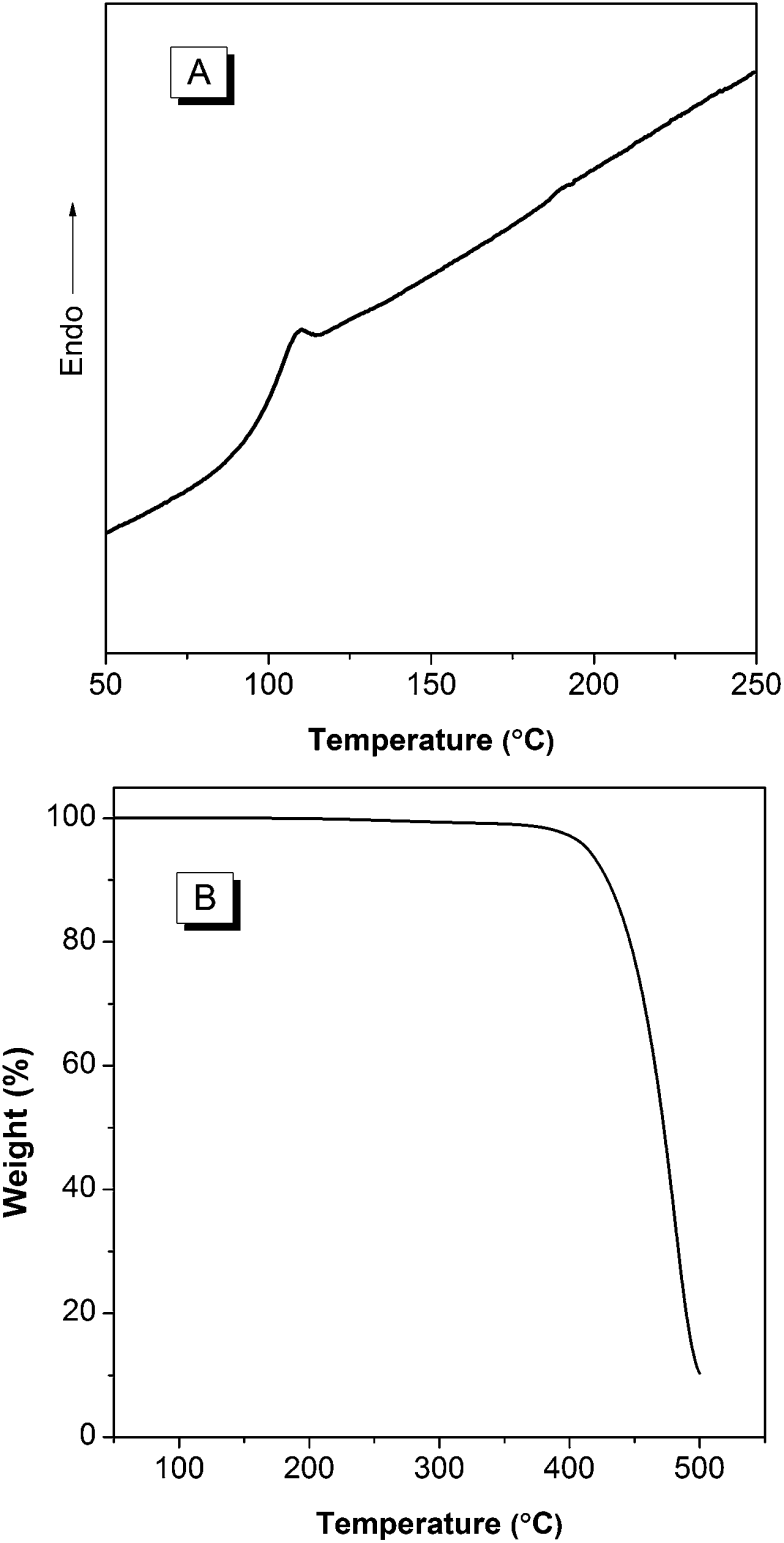
(A) DSC (second heating cycle) and (B) TGA thermogram of BP2TPAN recorded under nitrogen at scan rates of 5 °C min^−1^, respectively.

Excellent thermal stability is highly desired for the device fabrication and optoelectronic applications of the molecular conjugates. Particularly, *T*_g_ of an organic luminophore is one of the most important factors that influence the device stability and lifetime.^19^ When device is heated above *T*_g_ of the organic luminophore, an irreversible failure can occur. Therefore, we checked the thermal property by measuring TGA and DSC of BP2TPAN. As shown in Fig. 4, BP2TPAN has high thermal stability with its *T*_d_ (defined as the temperature at which a sample loses its 5% weight) value 414 °C. DSC analysis reveals the *T*_g_ of BP2TPAN is 96 °C, thus suggesting their exceptional thermal stability. Such outstanding thermal properties and efficient solid-state emissions render its application in optoelectronic devices.

### 3.5 OLED

**Fig. 5:**
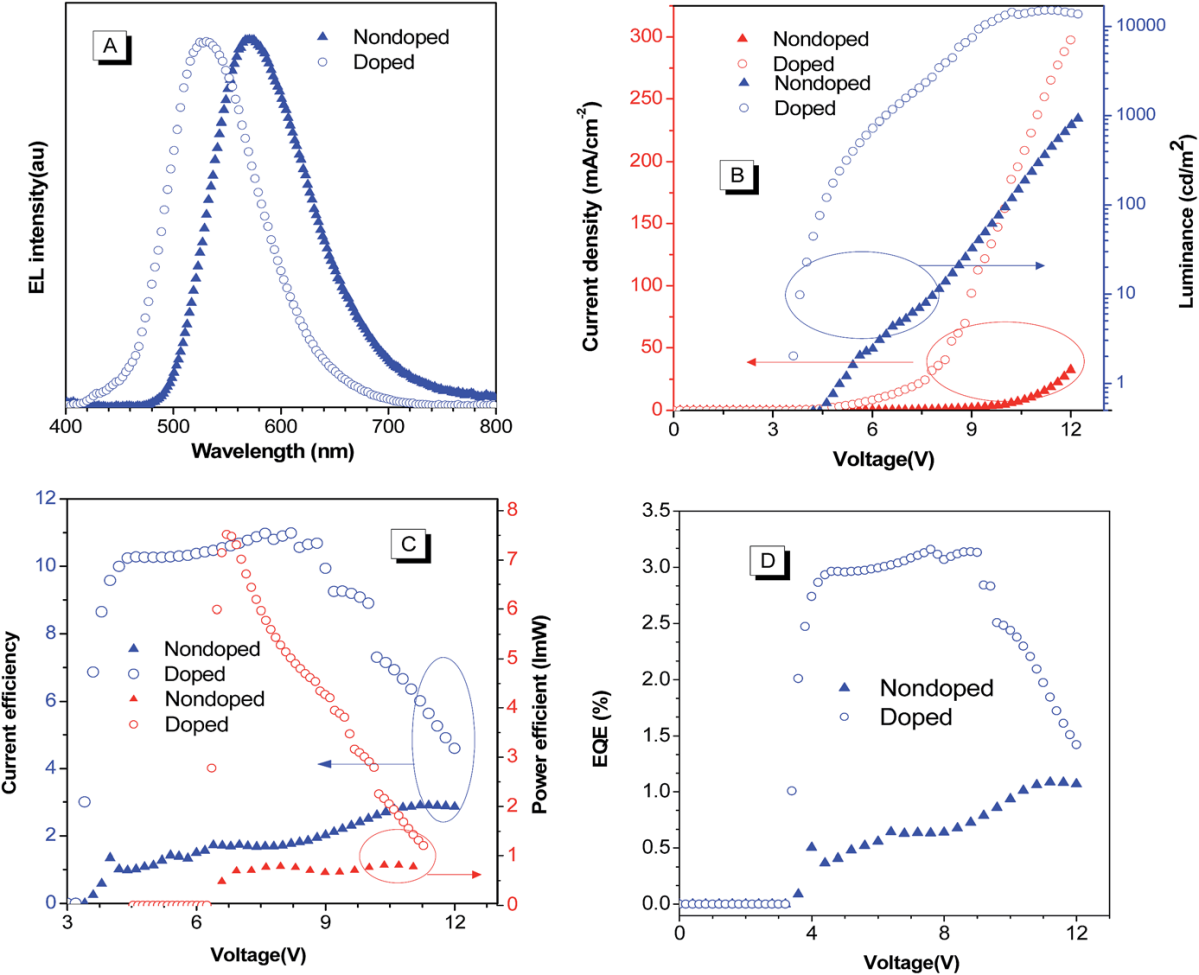
(A) EL spectra of BPA2TPAN doped and nondoped OLED devices (B) current efficiency–voltage–luminance, (C) current efficiency, power efficiency, and (D) external quantum efficiency *vs.* driving voltage of the devices with a general configuration of ITO/NPB/X/Bphen/LiF/Al, X = BP2TPAN (device I), BP2TPAN (3%)-MADN (device II).

The high solid-state emission efficiency and good thermal properties of BP2TPAN prompted us to explore their potential application in OLED devices. We first fabricated multilayer non-doped OLEDs with a general device configuration of ITO/NPB (50 nm)/BP2TPAN (20 nm)/Bphen (40 nm)/LiF (1 nm)/Al (100 nm), where NPB and Bphen were chosen as hole- and electron-transporting layers, respectively. The electroluminescence (EL) spectrum, current density–voltage–luminance characteristics, current efficiency, power efficiency and external quantum efficiency of the devices are shown in Fig. 5 and S6† and summarized in Table 1. The nondoped devices (I), emit orange light with EL maxima (*λ*_EL_) at 570 nm which are rather close to the PL emissions of their ground powders (580 nm). The device performance was moderate, with turn on voltages (*V*_on_), maximum luminance (*L*_max_), current efficiency (CE_max_), power efficiency (PE_max_), external quantum efficiency (EQE), and commission Internationale de L'Eclairage (CIE) being 4.8 V, 925 cd m^−1^, 2.9 cd A^−1^, 0.84 lm W^−1^, 1.1% and CIE (0.48, 0.51), respectively. Moreover, interestingly, when BP2TPAN doped into 2-methyl-9,10-di(2-naphthyl)anthracene (MADN) at a doping level of 3% (wt%) as an emitting layer, the device performance was dramatically improved by several times with *V*_on_, *L*_max_, CE_max_, PE_max,_ EQE and CIE are 3.2 V, 15 070 cd m^−1^, 11.0 cd A^−1^, 7.5 lm W^−1^, 3.1% and CIE (0.32, 0.55) (Fig. 5, Table 1 and Fig. S6 ESI†), respectively. Such considerably enhanced EL performance for the doped devices should be ascribed to the better matching of the energy gaps of each layer and thus a more effective charge balance in the devices. These results also suggest the feasibility of regulating the EL color through device technics.

**Table 1:** OLED device performances of BP2TPAN^a^

Device	*V*_on_, V	*λ*_max_, nm	*L*_max_, cd m^−2^	CE_max_, cd A^−1^	PE_max_, lm W^−1^	EQE, %	CIE, *x*, *y*
I	4.8	570	925	2.9	0.84	1.1	0.48, 0.51
II	3.2	530	15 070	11.0	7.5	3.1	0.32, 0.55

a Device configuration: ITO/NPB (50 nm)/X (20 nm)/Bphen (40 nm)/LiF (1 nm)/Al (100 nm); X = BP2TPAN (device I), BP2TPAN (3%)-MADN (device II); abbreviations: *V*_on_ = turn on voltages, *λ*_max_ = EL peak, *L*_max_ = maximal luminance, PE_max_ = maximal power efficiency, CE_max_ = maximal current efficiency, EQE = maximal quantum efficiency, CIE = Commission Internationale de L'Eclairage.

Indeed, the *λ*_EL_ values of the resulting doped and nondoped OLED devices were slightly blue shifted about 10 nm compared to ground and as prepared samples. This may be caused by under thermal evaporation, the molecule adopted more twisted configuration, and decreased conjugation length made EL spectrum slightly blue shifted.^30,31^

## 4 Conclusions

In summary, a novel triphenylacrylonitrile based BP2TPAN, whose highly twisted configurations are AIE luminogens, was successfully synthesized and applied for the mechanochromism and OLED applications. This AIE luminogens show high thermal stabilities, with their *T*_d_ and *T*_g_ values are 414 and 96 °C, respectively. The emission peaks, photoluminescence quantum yields and lifetimes of BP2TPAN and its ground sample are 540 and 580 nm, 74.3 and 8.4%, 3.4 and 5.1 ns, respectively. The non-optimized OLED non-doped device of the luminogens with *V*_on_, *L*_max_, CE_max_, PE_max_ and EQE are 4.8 V, 925 cd m^−2^, 2.9 cd A^−1^, 0.84 lm W^−1^, and 1.1%, respectively, giving yellow light (570 nm) EL with moderate efficiencies. Notably, the doped OLEDs devices emit significantly blue shifted yellow emissions, (530 nm) with considerably enhanced performance, whose *V*_on_, *L*_max_, CE_max_, PE_max_ and EQE are 3.2 V, 15 070 cd m^−2^, 11.0 cd A^−1^, 7.5 lm W^−1^, and 3.1%, respectively. The AIE feature, high solid-state efficiency, mechanochromic properties, excellent thermal stability, and bicolor EL render BP2TPAN as a highly promising candidate for versatile optoelectronic applications.

## Conflicts of interest

There are no conflicts to declare.

## Supplementary Material

RA-008-C7RA10174K-s001
