# Supplementary material for: A novel triphenylacrylonitrile based AIEgen for high contrast mechanchromism and bicolor electroluminescence
Source: RSC Adv. 2018 Jan 2;8(2):710–6. doi: 10.1039/c7ra10174k (PMC9076843; doi:10.1039/c7ra10174k)
Supplement: RA-008-C7RA10174K-s001 [file RA-008-C7RA10174K-s001.pdf]

Electronic Supplementary Material (ESI) for RSC Advances.
This journal is © The Royal Society of Chemistry 2018

Electronic Supporting Information

A novel triphenylacrylonitrile based AIEgen for high contrast mechanchromism and bicolor electroluminescence

Jiayou Hu[a], Bingli Jiang[b], Yongyang Gong[a]*, Yuanli Liu[a], Gang He[a], Wang Zhang Yuan[c]* and Chun Wei[a]*

[a]*Key Laboratory of New Processing Technology for Non-ferrous Metals and Materials - Ministry of Education, GuangXi Key Laboratory of New Energy and Building Energy saving, College of Materials Science and Engineering, Guilin University of Technology, Guilin, 541004, China*

[b] *College of Pharmacy, Guilin Medical University , Guilin 541004, China*

[c] *School of Chemistry and Chemical Engineering, Shanghai Jiao Tong University, Shanghai 200240, China*

Table of Contents

Fig. S1 Synthetic routes of BP2TPAN.

Fig. S2 Photographs of isolated BP2TPAN on TLC plate under 254 nm UV light irradiation after being developed in 1/1 petroleum ether-Chloroform mixture.

Fig. S3 ^{1}H-NMR spectrum of BP2TPAN in THF-d_8.

Fig. S4 HRMS spectrum of BP2TPAN.

Fig. S5 UV-Vis spectrum of BP2TPAN in THF.

Fig.S6 Commission Internationale de L'Eclairage coordinates of the OLED doped and undoped devices of BPA2TPAN.

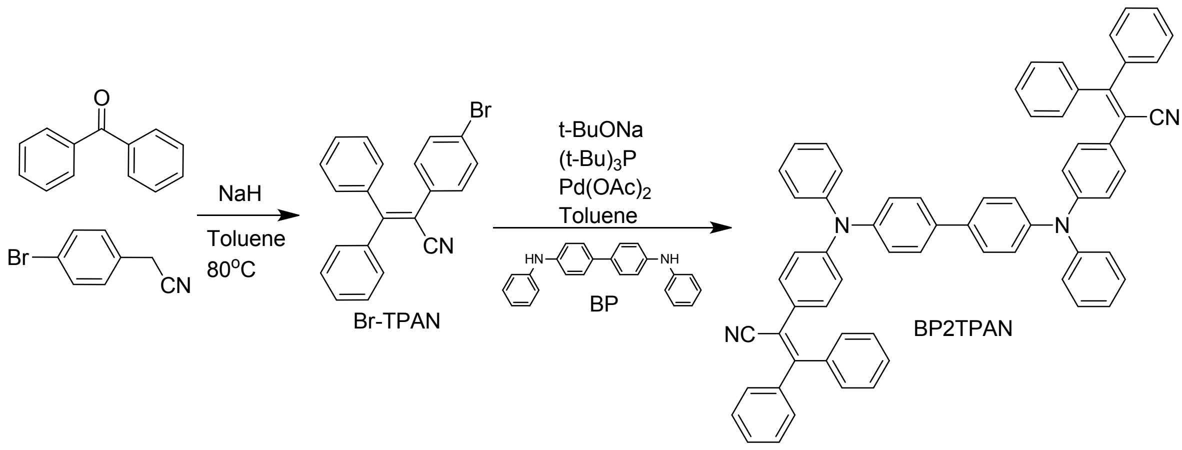

Fig.S1 Synthetic routes of BP2TPAN.

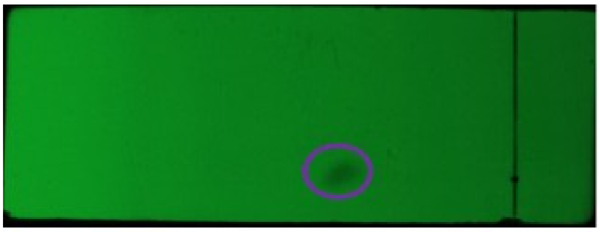

Fig. S2 Photographs of isolated BP2TPAN on TLC plate under 254 nm UV light irradiation after being developed in 1/1 petroleum ether-Chloroform mixture.

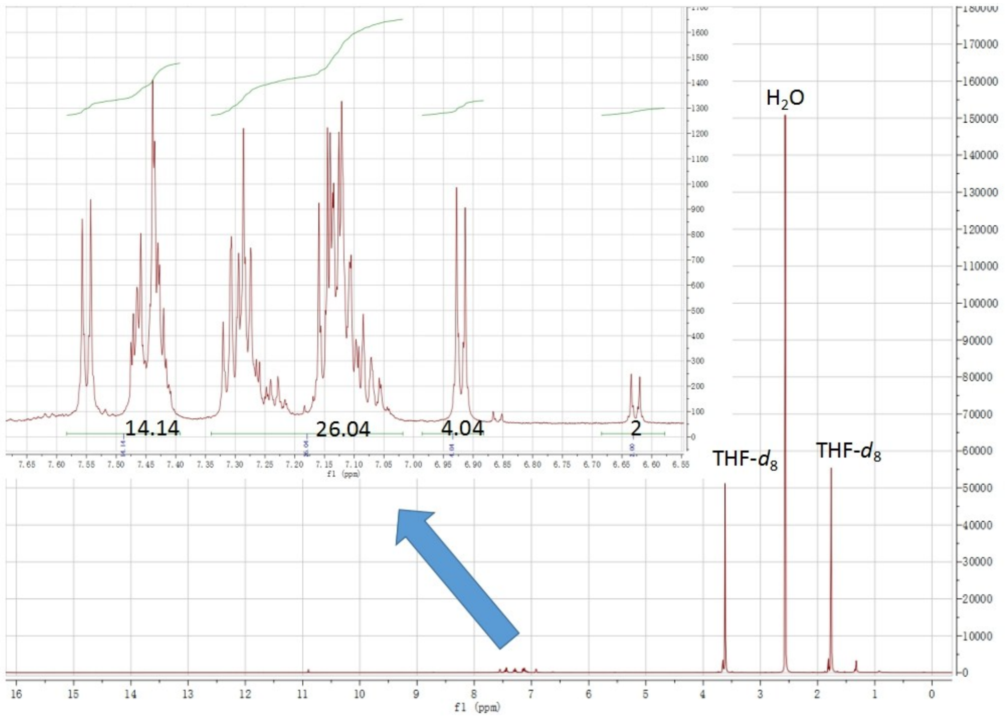

Fig. S3 ^{1}H-NMR spectrum of BP2TPAN in THF-d_8.

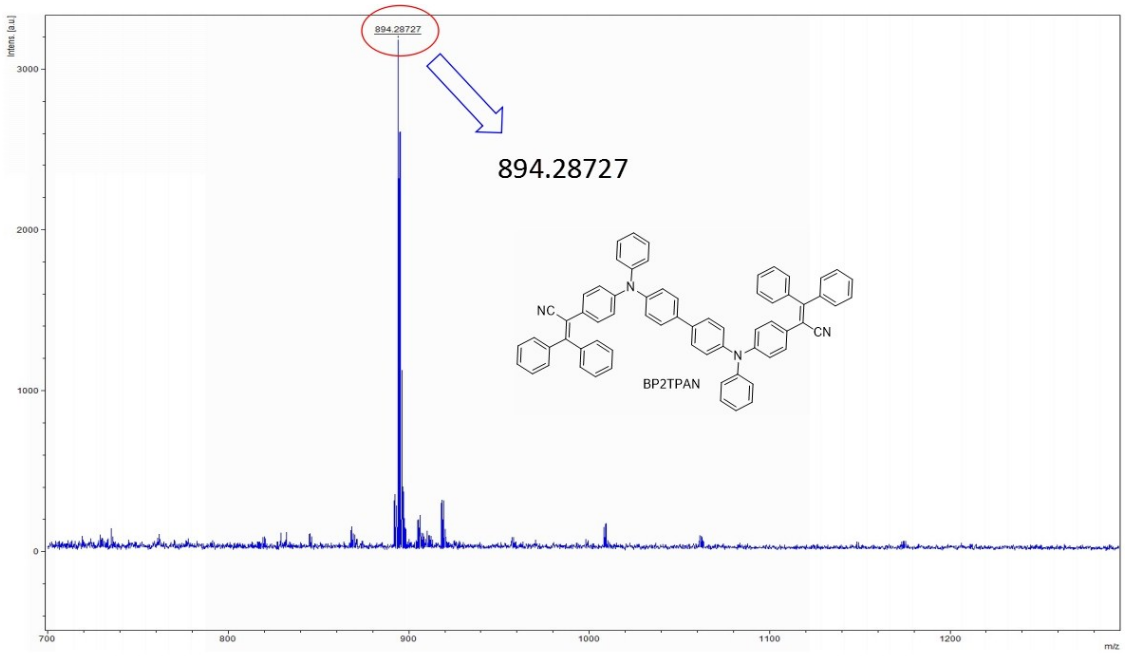

Fig. S4 HRMS spectrum of BP2TPAN.

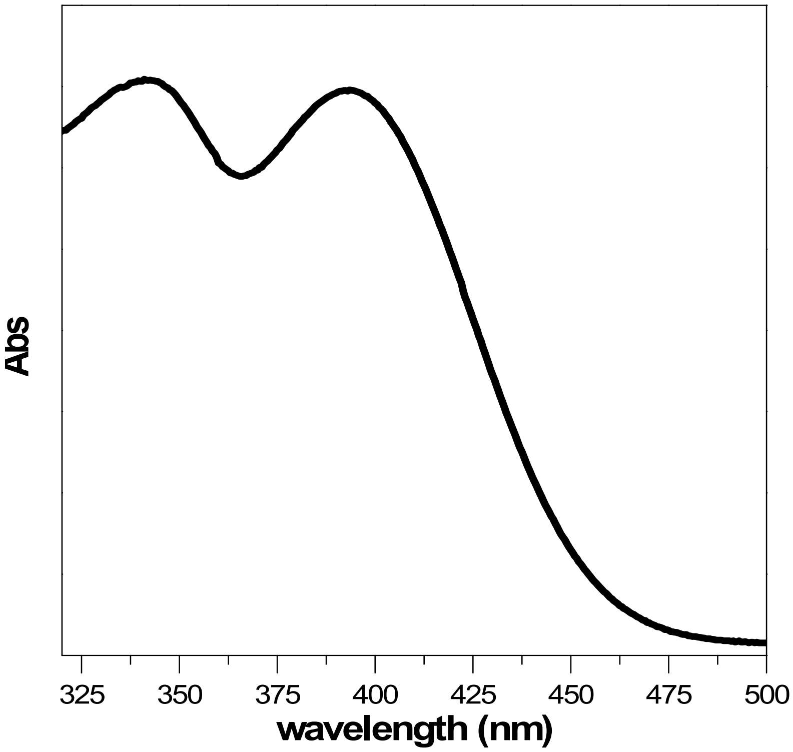

Fig. S5 UV-Vis spectrum of BP2TPAN in THF. Concentration = 5 μM.

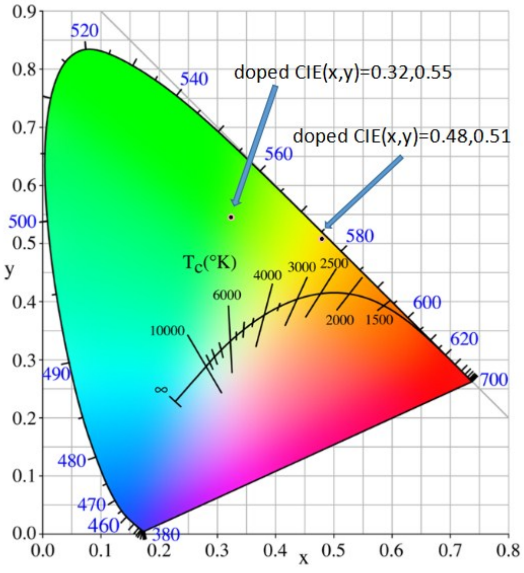

Fig.S6 Commission Internationale de L'Eclairage coordinates of the OLED doped and nondoped devices of BPA2TPAN.
